# Acute kidney injury after cardiac arrest

**DOI:** 10.1186/s13054-015-0900-2

**Published:** 2015-04-17

**Authors:** Omar Tujjar, Giulia Mineo, Antonio Dell’Anna, Belen Poyatos-Robles, Katia Donadello, Sabino Scolletta, Jean-Louis Vincent, Fabio Silvio Taccone

**Affiliations:** Department of Intensive Care, Erasme Hospital, Université Libre de Bruxelles (ULB), Route de Lennik, 808, Brussels, 1070, Belgium

## Abstract

**Introduction:**

The aim of this study was to evaluate the incidence and determinants of AKI in a large cohort of cardiac arrest patients.

**Methods:**

We reviewed all patients admitted, for at least 48 hours, to our Dept. of Intensive Care after CA between January 2008 and October 2012. AKI was defined as oligo-anuria (daily urine output <0.5 ml/kg/h) and/or an increase in serum creatinine (≥0.3 mg/dl from admission value within 48 hours or a 1.5 time from baseline level). Demographics, comorbidities, CA details, and ICU interventions were recorded. Neurological outcome was assessed at 3 months using the Cerebral Performance Category scale (CPC 1–2 = favorable outcome; 3–5 = poor outcome).

**Results:**

A total of 199 patients were included, 85 (43%) of whom developed AKI during the ICU stay. Independent predictors of AKI development were older age, chronic renal disease, higher dose of epinephrine, in-hospital CA, presence of shock during the ICU stay, a low creatinine clearance (CrCl) on admission and a high cumulative fluid balance at 48 hours. Patients with AKI had higher hospital mortality (55/85 vs. 57/114, p = 0.04), but AKI was not an independent predictor of poor 3-month neurological outcome.

**Conclusions:**

AKI occurred in more than 40% of patients after CA. These patients had more severe hemodynamic impairment and needed more aggressive ICU therapy; however the development of AKI did not influence neurological recovery.

## Introduction

Most studies on patients after resuscitated cardiac arrest (CA) have focused on survival or the extent of brain dysfunction [1]; the prevalence of extra-cerebral organ injury and its impact on outcome has been less well-characterized [2]. After return of spontaneous circulation (ROSC), myocardial dysfunction and the systemic ischemia/reperfusion response can lead to the so-called post cardiac arrest syndrome, which is characterized by the activation of immunologic and coagulation pathways and the release of inflammatory mediators, all leading to tissue hypoperfusion and multiple organ dysfunction [3].

Recently, Roberts *et al*. [4] reported that 96% of a cohort of 203 patients resuscitated after CA had some degree of organ dysfunction, in particular cardiovascular and respiratory impairment; two-thirds of these patients had at least two extra-cerebral organ dysfunctions. Only alterations in the cardiovascular and respiratory system, assessed by the sequential organ failure assessment (SOFA) subscores [5], were independently associated with in-hospital mortality.

Several studies have also highlighted the high prevalence of acute kidney injury (AKI) in CA patients and how this may influence patient outcome in this setting. Domanovits *et al*. [6] showed that 12% of CA patients developed AKI within 24 h after admission; in particular, congestive heart failure, history of hypertension and the total dose of epinephrine administered during cardiopulmonary resuscitation (CPR) were independent predictors of AKI. However, AKI was defined as a 25% decrease in creatinine clearance (CrCl) using the Cockroft-Gault formula, instead of the currently recommended combination of changes in serum creatinine and urine output [7]. In addition, this study was conducted before the implementation of therapeutic hypothermia (TH), which may be associated with enhanced protection of visceral organs [8]. Other studies report that AKI occurs in 30 to 50% of CA patients and is associated with high levels of biomarkers of brain injury, the presence of cardiogenic shock and inadequate fluid therapy [9-11]. Nevertheless, the association of AKI with survival remains unclear [9,12]. Moreover, these studies did not consider the presence of potentially nephrotoxic agents (for example, contrast media, aminoglycosides), the development of sepsis during the ICU stay, which can significantly increase the risk of AKI, and the amount and type of fluid administered after hospital admission, as potential risk factors. Augmented renal clearance (ARC) can also occur in critically ill patients and is associated with poor outcome, but the occurrence and the impact of ARC in CA patients have not been well-studied [13,14].

Finally, AKI may have an impact on neurological recovery. In an experimental model of renal injury, AKI was found to contribute to the inflammatory injury in the hippocampus, altered blood–brain barrier permeability and potentiated oxidative stress in the cerebral tissue [15,16]. Patients with AKI also have a higher incidence of stroke and cognitive disorders than patients without AKI [17,18]. Nevertheless, the association of AKI and long-term neurological recovery has not been well-studied in CA patients. The aim of this study was, therefore, to evaluate the incidence and the determinants of AKI in a large cohort of CA patients, as well as the occurrence of ARC and the association of changes in renal function with neurological outcome.

## Methods

### Study population

This retrospective study was performed in the Department of Intensive Care at Erasme Hospital, Brussels. The local Ethical Committee (*Comité d’Ethique Hospitalo-Facultaire Erasme-ULB*) approved the study, but waived the need for informed consent because the study was purely observational. The study was performed in accordance with the ethical standards of the 1964 Declaration of Helsinki and its later amendments. All comatose patients (Glasgow coma scale (GCS) score <9) admitted after in-hospital CA (IHCA) or out-of-hospital CA (OHCA) (January 2008 to October 2012) were included in an institutional database, provided that they survived for at least 48 h after CA, so that at least three serum creatinine (sCr) measurements were obtained. Exclusion criteria were missing data on creatinine or urine output, AKI (for IHCA) before the occurrence of CA and chronic renal failure requiring hemodialysis.

### Post-resuscitation care

All these patients were treated with TH, targeting a body temperature between 32 and 34°C for 24 h, according to a standardized institutional protocol, including the use of a cold fluid bolus (20 to 30 mL/kg saline or a balanced crystalloid solution over 30 minutes) and of a water-circulating blanket device (Medi-Therm II, Gaymar, Orchard Park, NY, USA). Midazolam (continuous infusion 0.03 to 0.1 mg/kg/h) and morphine (0.1 to 0.3 mg/kg/h) were administered during the hypothermic phase, while neuromuscular blocking agents (NMBAs, cisatracurium as a bolus of 0.15 mg/kg) were given in the induction phase and, if needed, as a continuous infusion thereafter (1 to 3 mcg/kg/min). Re-warming was performed passively at a maximum rate of 0.5°C/h; sedation, analgesia and NMBAs were discontinued when the body temperature exceeded 37°C.

Patients were kept in a 30° semi-recumbent position; ventilation was set to maintain arterial partial pressure of carbon dioxide (PaCO_2_) at 35–45 mmHg and peripheral capillary oxygen saturation (SpO2) >94%. Invasive hemodynamic monitoring (PiCCO, Pulsion, Munich, Germany) was placed whenever needed and transesophageal echocardiography was performed within the first 8 to 12 h after ICU admission in all patients. Mean arterial pressure (MAP) was maintained at >65 to 70 mmHg using fluids, dobutamine and/or norepinephrine, whenever needed. Higher levels of MAP were targeted in patients with a history of hypertension and in patients with low (<60%) cerebral oximetry values (Foresight, CasMed, Branford, CT, USA). Intra-aortic balloon counterpulsation (IABP) or extracorporeal membrane oxygenation (ECMO) was initiated in cases of severe cardiogenic shock. A local insulin protocol was applied to keep blood glucose levels between 110 and 150 mg/dL in all patients.

After re-warming the patient and stopping sedative agents, repeated neurologic examination and standard or continuous electroencephalogram (EEG) were performed. Withdrawal of life-support was an interdisciplinary decision based on bilateral absence of the N20 response to somatosensory evoked potentials (SSEPs), persisting deep coma, or presence of status myoclonus or refractory status epilepticus.

### Data collection

We collected data on demographics, pre-existing chronic diseases and CPR (that is, first rhythm, bystander CPR, time to ROSC, total epinephrine dose) in all patients. Serum creatinine levels on ICU admission were recorded, as was the CrCl on the first day of therapy, calculated from the 24-h urine collection, according to the following formula:

$$ \begin{array}{l}\mathrm{C}\mathrm{r}\mathrm{C}\mathrm{l},\mathrm{mL}/ \min = \left(\left(\mathrm{Urine}\ \mathrm{output},\mathrm{mL}\right)\ast \left(\mathrm{Urine}\ \mathrm{creatinine},\mathrm{mg}/\mathrm{dL}\right)\right)/\\ {}\left[\left(\mathrm{s}\mathrm{C}\mathrm{r},\mathrm{mg}/\mathrm{dL}\right)\ast \left(\mathrm{Urine}\ \mathrm{collection}, \min \right)\right).\end{array} $$

sCr and urine output were collected daily during the entire ICU stay. Daily fluid balance and the administration of hydroxyethyl starch (HES) solutions were also noted. We also recorded use of any potentially nephrotoxic agents, including intravenous contrast media, angiotensin-converting enzyme inhibitors (ACEIs), angiotensin II receptor blockers (ARBs), non-steroidal anti-inflammatory drugs (NSAIDs), aminoglycosides, amphotericin B, colistin and immunosuppressant agents (for example, calcineurin inhibitors, such as cyclosporine and tacrolimus). Treatments with vasoactive drugs, mechanical ventilation, and continuous renal replacement therapy (CRRT) were recorded, as were length of ICU stay and ICU and hospital outcome. Neurological evaluation at 3 months after CA was assessed using the cerebral performance category score (CPC; 1 = no neurological disability, 2 = mild neurological disability, 3 = severe neurological impairment, 4 = vegetative state, 5 = death). The CPC evaluation was assessed during follow-up visits or by telephone interview with the general practitioner.

### Definitions

AKI was defined as a daily urine output <0.5 mL/kg/h and/or an increase in sCr level by at least 0.3 mg/dL or >1.5 time increase from baseline values [19]. In particular, the criterion of ≥0.3 mg/dL rise from baseline values occurred within 48 h and was not used with any previous chronic renal disease or in the absence of baseline sCr. We considered only daily urine output, because this measure may be influenced less by the effects of specific therapies, such as volume loading and diuretic administration, compared to shorter periods. Baseline sCr was collected from the patient’s medical records within one year prior to the CA (if multiple values were available, the last one was collected). When a baseline sCr was not available (n = 10, all with OHCA), it was estimated using the Modification of diet in renal disease (MDRD) study equation, which provides sCr values based on the glomerular filtration rate calculated by age, race and sex [20]. Chronic kidney disease (CKD) was identified as CKD stage 3 according to the recent definitions [21]. For patients with CKD and those with estimated baseline sCr, the criterion of sCr increase >1.5 times the baseline value was used. The more severe AKI stage during the ICU stay [19] as well as the time to AKI since ICU admission was also noted. ARC was defined as a CrCl of 130 mL/min or greater [22]. Shock was defined as the need for vasopressor agents for more than 6 h. The diagnosis of infection was based on the combination of clinical or radiological signs of an infectious process with a positive culture from a normally sterile site (for example, blood culture, bronchoalveolar lavage (BAL) or endotracheal aspirate) and the need for antibiotic administration. The site of infection was defined using the CDC/NHSN criteria [23]. A favorable neurological outcome was defined as a CPC of 1 or 2 at 3 months; poor neurological outcome as a CPC of 3 to 5.

### Statistical analysis

Statistical analyses were performed using the SPSS 18.0 for Windows NT software package (SPSS Inc. 2004, Chicago, IL, USA). Descriptive statistics were computed for all study variables. The Kolmogorov-Smirnov test was used, and histograms and normal-quantile plots were examined to verify the normality of distribution of continuous variables. Discrete variables were expressed as counts (percentage) and continuous variables as means ± SD or median (25th to 75th percentiles). Demographics and clinical differences between groups (AKI versus no AKI; favorable versus poor neurological outcome) were assessed using the chi-square test, Fisher’s exact test, Student’s *t*-test, or Mann–Whitney *U*-test, as appropriate. The significance of differences in sCr between groups was analyzed using two-way (time and group) analysis of variance for repeated measures (ANOVA), followed by Bonferroni post-hoc analysis. Multivariable logistic regression analysis with AKI development as the dependent variable was performed in all patients. Colinearity between variables was excluded prior to modelling; only variables associated with a higher risk of AKI (*P* <0.2) on a univariate basis were introduced in the multivariate model. Odds ratios (OR) with 95% confidence intervals (CI) were computed. The same analysis was then performed to identify independent predictors of favorable neurological outcome. A *P*-value <0.05 was considered as statistically significant.

## Results

**Figure 1:**
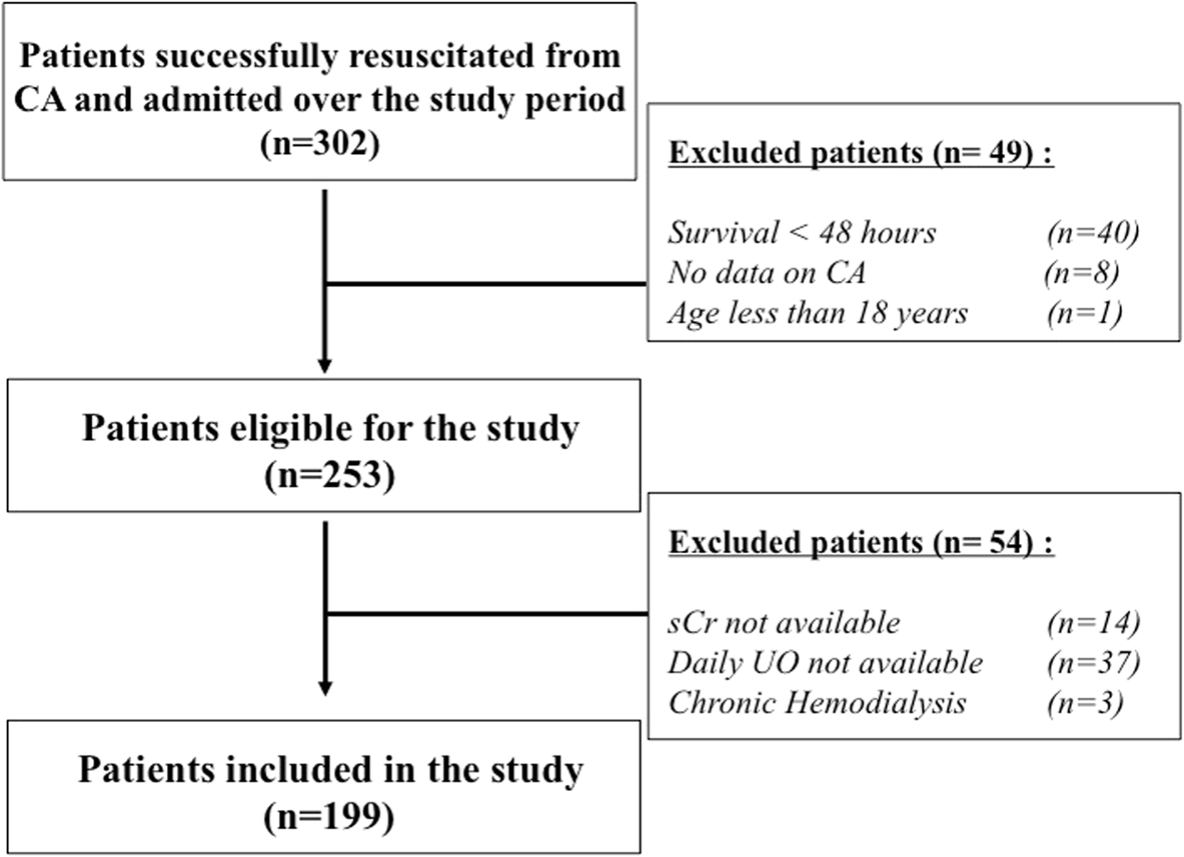
**Flow-chart of the study.** sCr, serum creatinine; CA, cardiac arrest; UO, urine output.

Of the 253 CA patients eligible over the study period, 54 were excluded (n = 51 for missing data on sCr/urine output; n = 3 because they were receiving chronic hemodialysis - hospital mortality 29/51 (57%) and favorable neurological outcome 19/51 (37%)) and 199 were included in the final cohort (Figure 1). The main characteristics of the study population are shown in Table 1. Sixty-nine patients had CA of non-cardiac origin (respiratory failure with severe hypoxemia, n =45; septic shock, n = 16; drug overdose, n = 5; trauma, n = 1; drowning, n = 1; hanging, n = 1). Overall ICU survival was 46% and 81 patients (41%) had a favorable neurological outcome at 3 months.

**Table 1:** **Characteristics of the study population according to the development of acute kidney injury (AKI)**

	**All patients (n = 199)**	**No AKI (n = 114)**	**AKI (n = 85)**	***P*** **-** ***value***
Age, years	62 ± 16	60 ± 14	65 ± 10	0.001
Weight, kg	77 ± 22	76 ± 22	78 ± 20	0.45
Male, n (%)	134 (67)	80 (70)	54 (64)	0.29
Total ICU stay, days	4 (3 to 8)	4 (3 to 7)	5 (3 to 9)	0.03
Witnessed CA, n (%)	148 (74)	77 (68)	71 (84)	0.009
Bystander CPR, n (%)	112 (56)	63 (55)	49 (58)	0.67
Time to ROSC, minutes	15 (9 to 26)	15 (9 to 22)	16 (10 to 30)	0.04
Epinephrine, mg	4 (2 to 7)	4 (2 to 6)	4 (3 to 8)	0.03
Out-of-hospital CA	121 (61)	80 (70)	41 (48)	0.002
Non cardiac-origin CA	69 (35)	44 (39)	25 (29)	0.22
VF/VT, n (%)	118 (59)	48 (42)	33 (39)	0.66
**Past history**				
Chronic heart failure, n (%)	57 (29)	25 (22)	32 (38)	0.02
Hypertension, n (%)	77 (39)	42 (37)	35 (41)	0.56
Coronary artery disease, n (%)	86 (43)	45 (39)	41 (48)	0.25
Diabetes, n (%)	42 (21)	19 (17)	23 (27)	0.08
COPD/asthma, n (%)	37 (19)	24 (21)	13 (15)	0.36
Neurological disease, n (%)	30 (15)	22 (19)	8 (9)	0.07
Chronic renal disease, n (%)	30 (15)	9 (8)	21 (25)	0.001
Liver cirrhosis, n (%)	11 (6)	4 (4)	7 (8)	0.13
HIV, n (%)	1 (1)	1 (1)	0	1.00
Corticosteroid therapy, n (%)	29 (15)	12 (11)	17 (20)	0.07
Immunosuppressive agents, n (%)	2 (1)	0	2 (2.3)	0.09
**Therapy before ICU admission**				
IECA/ ARBs, n (%)	81 (41)	43 (38)	38 (45)	0.38
Diuretics, n (%)	40 (20)	15 (13)	25 (29)	0.008
Aminoglycosides, n (%)	1 (1)	0	1 (1)	0.21
Amphotericin B, n (%)	0	0	0	-
Cyclosporine/FK506, n (%)	0	0	0	-
NSAIDs, n (%)	68 (34)	37 (32)	31 (36)	0.58
Chemotherapy, n (%)	0	0	0	-
**Events and interventions during ICU stay**				
Lactate on admission, mEq/L	3.1 (1.9 to 5.1)	3.1 (1.8 to 5.2)	3.3 (2.0 to 6.6)	0.288
Infection, n (%)	123 (62)	66 (58)	57 (67)	0.21
IABP, n (%)	15 (8)	6 (5)	9 (11)	0.12
ECMO, n (%)	22 (11)	9 (8)	13 (16)	0.08
Shock, n (%)	103 (52)	43 (38)	60 (71)	0.002
Therapeutic hypothermia, n (%)	198 (99)	114 (100)	84 (99)	0.96
MV, n (%)	199 (100)	114 (100)	85 (100)	-
Duration of MV, days	3 (2 to 5)	3 (2 to 4)	4 (3 to 7)	0.001
Fluid balance on day 1, mL/day	1,924 ± 1348	1,480 ± 1348	2,520 ± 1414	0.001
Fluid balance on day 2, mL/day	2,704 ± 1636	2,282 ± 1639	3,264 ± 2889	0.003
CrCl on day 1, mL/min	40 (13 to 90)	67 (27 to 121)	23 (9 to 44)	<0.001
Augmented renal clearance on day 1, n (%)	38 (19)	32 (28)	6 (7)	0.001
CRRT, n (%)	24 (12)	0	24 (29)	0.001
**Therapy during ICU stay**				
Vasopressor therapy, n (%)	134 (68)	65 (57)	69 (81)	0.001
Duration of vasopressor therapy, days	3 (2 to 5)	3 (2 to 4)	4 (3 to 6)	0.001
Cumulative vasopressor dose, mcg	21.0 (7.7-73.2)	9.9 (4.1-29.9)	39.1 (14.6-109.7)	0.001
Dobutamine therapy, n (%)	112 (56)	40 (35)	72 (85)	0.001
Duration of dobutamine, days	3.5 (3 to 6)	3 (2 to 4)	4 (3 to 6)	0.001
Cumulative dobutamine dose, mcg	1456 (514 to 2733)	982 (445 to 1670)	2084 (575 to 3964)	0.001
ACEIs/ARBs, n (%)	54 (27)	36 (32)	18 (21)	0.11
Diuretics, n (%)	94 (47)	45 (40)	49 (57)	0.01
Aminoglycosides, n (%)	38 (19)	15 (13)	23 (27)	0.01
Amphotericin B, n (%)	1 (1)	1 (1)	0	-
Cyclosporine/FK506, n (%)	2 (1)	0	2 (2)	0.92
NSAIDs, n (%)	90 (45)	49 (43)	41 (48)	0.48
Chemotherapy, n (%)	2 (1)	0	2 (2)	0.86
Contrast medium, n (%)	94 (47)	51 (45)	43 (51)	0.41
Contrast medium injections, n (%)	0 (0 to 1)	0 (0 to 1)	1 (0 to 2)	0.19
HES, n (%)	14 (7)	8 (7)	6 (7)	0.99
Number of nephrotoxic agents, n (%)	2 (1 to 3)	1 (0 to 3)	2 (1 to 3)	0.04
At least one nephrotoxic agent, n (%)	158 (79)	83 (72)	75 (88)	0.008
**Outcomes**				
ICU mortality, n (%)	107 (54)	55 (48)	52 (61)	0.08
Hospital mortality, n (%)	112 (56)	57 (50)	55 (65)	0.04
Favorable neurological outcome at 3 months, n (%)	81 (41)	52 (46)	29 (34)	0.11

Results are expressed as mean ± SD, median (IQR) or number (%). CA, cardiac arrest; CPR, cardiopulmonary resuscitation; ROSC, return of spontaneous circulation; VF/VT, ventricular fibrillation/ventricular tachycardia; COPD, chronic obstructive pulmonary disease; IABP, intra-aortic balloon pump counterpulsation; ECMO, extracorporeal membrane oxygenation; CRRT, continuous renal replacement therapy; ACEIs, angiotensin converting enzyme inhibitors; ARBs, angiotensin II receptor blockers; NSAIDs, non-steroidal anti-inflammatory drugs; HES, hydroxyethyl starch; CrCl, creatinine clearance; MV, mechanical ventilation.

**Figure 2:**
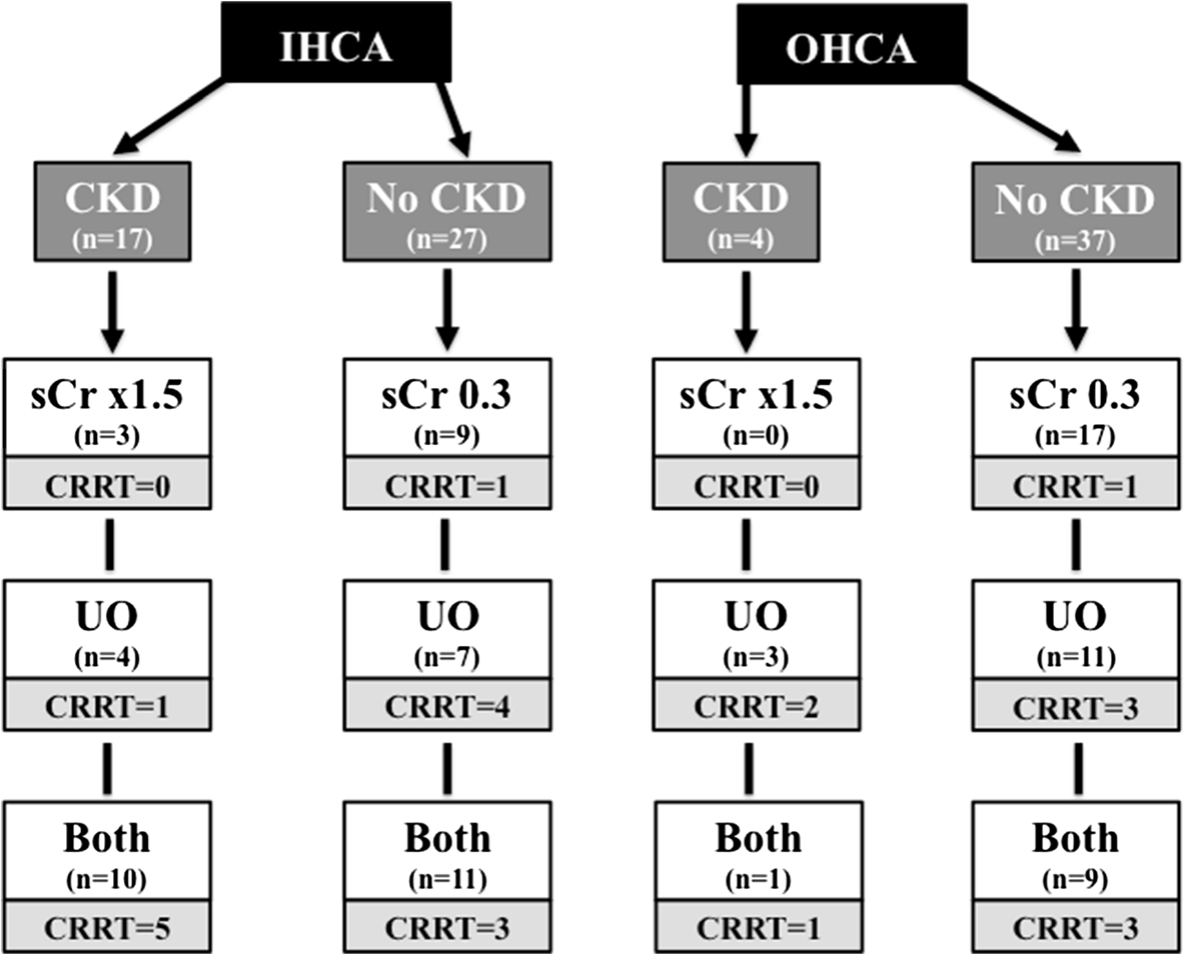
**Diagnosis of acute kidney injury (AKI) in patients suffering from out-of-hospital cardiac arrest (OHCA) or in-hospital (IHCA) cardiac arrest.** Among patients with previous chronic renal disease (CKD), diagnosis of AKI was initially based on the increase of serum creatinine (sCr) of at least 1.5 times the baseline values or the reduction in daily urine output (UO) or both. Among patients without previous chronic renal disease (No CKD), diagnosis of AKI was initially based on the increase of serum creatinine (sCr) ≥0.3 mg/dL from the baseline value or the reduction of daily urine or both. For each group, the number of patients eventually treated with continuous renal replacement therapy (CRRT) was reported.

**Figure 3:**
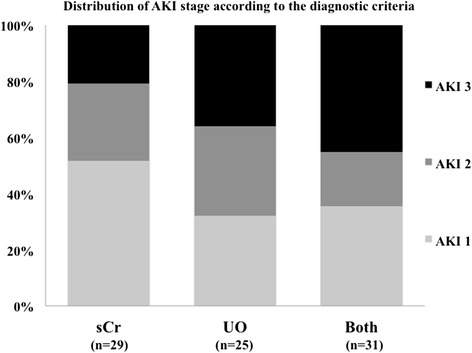
**Proportion of patients developing acute kidney injury (AKI) stage 1, 2 or 3 according to the diagnosis of AKI based on the serum creatinine (sCr) criterion, the urine output (UO) criterion or both.**

Eighty-five patients (43%) developed AKI, after a median of 2 (2, 3) days; CKD was found in 21 patients (25% of the AKI patients) and CRRT was used in 24 patients (29% of the AKI patients) (Figure 2). A total of 34 patients (40%) developed AKI stage 1, 22 (26%) AKI stage 2 and 29 (34%) AKI stage 3; the distribution of AKI stages was similar according to the initial criterion of AKI diagnosis (Figure 3; *P* = 0.19). Patients who developed AKI were significantly older than those who did not, were more likely to have chronic heart failure, pre-existing neurological or renal disease, and to be already receiving steroid or diuretic therapy. Patients who developed AKI also more frequently had a witnessed and IHCA than other patients, related to the larger number of witnessed CA among patients with IHCA than OHCA patients (73/78 versus 75/121, *P* <0.001). Patients who developed AKI had a longer time to ROSC and received more epinephrine during CPR than other patients. These patients were also more likely to have shock, to be treated with vasopressors and dobutamine, and to have a longer duration of mechanical ventilation during the ICU stay than those who did not develop AKI. Finally, patients who developed AKI received more nephrotoxic agents, in particular aminoglycosides, had a greater fluid balance over the first 2 days after ICU admission and were more likely to receive diuretics during the ICU stay. Patients who developed AKI had greater hospital mortality (65% versus 50%, p = 0.04) than those who did not and there was a trend towards fewer patients with a favorable neurological outcome at 3 months (34% versus 46%, *P* = 0.08). Thirty-eight patients (19%) had ARC on the first day after ICU admission, and six of them eventually developed AKI, after a median of 3 (range 2 to 5] days; only one patient needed CRRT.

In multivariable regression analysis, older age, larger epinephrine dose during CPR, the occurrence of shock during the ICU stay, higher 48-h fluid balance and chronic renal disease were independent predictors of AKI after CA. Augmented CrCl on the first day of the ICU stay and OHCA were protective factors against AKI development (Table 2).

**Table 2:** **Multivariable analysis to identify the independent predictors of development of acute kidney injury after cardiac arrest**

**Variable**	***P*** **-value**	**Odds ratio**	**95% CI for odds ratio**	
			**Lower**	**Upper**
Age, years	0.027	1.030	1.003	1.057
Epinephrine, mg	0.004	1.198	1.059	1.356
Out-of-hospital cardiac arrest	0.006	0.289	0.118	0.706
Shock during ICU stay	0.026	2.855	1.133	7.195
Chronic renal disease	0.024	4.466	1.222	16.318
Fluid balance at 48 h, L	0.047	1.120	1.020	1.200
Creatinine clearance on day 1, mL/min	0.015	0.991	0.984	0.998

**Figure 4:**
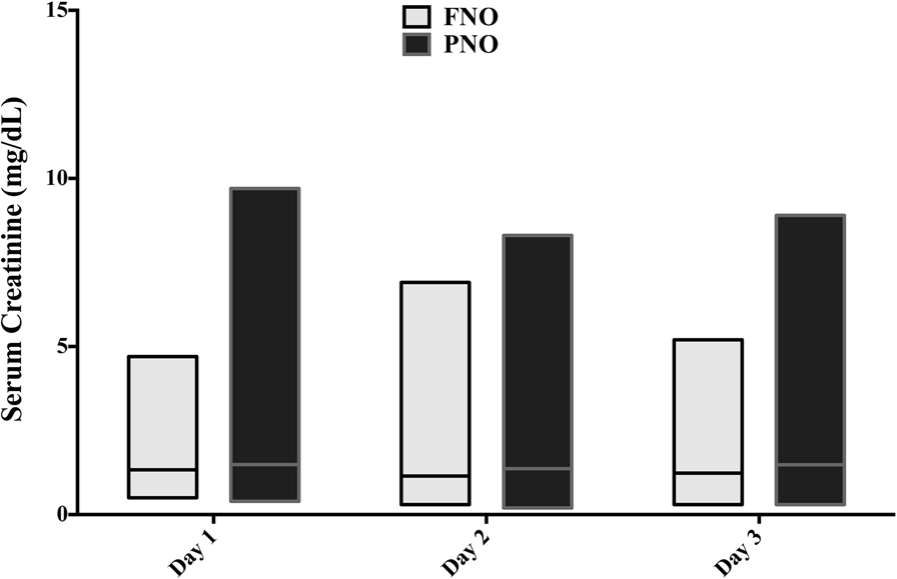
**Time course of median serum creatinine over the first 3 days following ICU admission in patients with favorable (FNO) and poor (PNO) neurological outcomes.**

**Figure 5:**
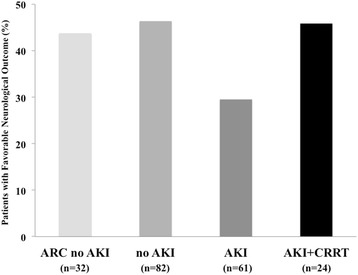
**Proportion of patients with favorable neurological outcome according to the presence of augmented renal clearance (ARC) without acute kidney injury (AKI), normal renal function (no AKI), AKI or AKI requiring continuous renal replacement therapy (CRRT).**

Patients with a favorable 3-month neurological outcome were younger, more frequently male, and less likely to have a history of heart failure than those with poor neurological outcome; they had also more frequently had a witnessed CA, bystander CPR, initial shockable rhythm, lower epinephrine dose, CA of non-cardiac origin, shorter time to ROSC, less commonly had shock during the ICU stay, had a lower blood lactate level on admission and required lower doses of vasopressors and dobutamine. There were no significant differences in sCr levels during the first 3 days of ICU admission between patients with favorable and poor neurological outcomes (Figure 4). Hospital mortality was higher, although not statistically significant, for those patients diagnosed with AKI using sCr or combined sCr/UO criteria than in those diagnosed by UO alone (20/29 versus 22/13 versus 13/25, respectively; *P* = 0.28). Similar results were found for a 3-month favorable neurological outcome (8/28 versus 9/31 versus 12/25, respectively, *P* = 0.24). The development of ARC, AKI or need for CRRT had no significant impact on neurological outcome (Figure 5). In a multivariable analysis, younger age, shockable rhythm, bystander CPR, a shorter time to ROSC, lower dose of epinephrine during CPR, the absence of shock during the ICU stay and lower blood lactate level on ICU admission were independent factors associated with a favorable 3-month neurological outcome (Table 3).

**Table 3:** **Univariable and multivariable analyses to assess variables independently associated with favorable neurological outcome at 3 months**

**Variable**	**Favorable neurological outcome (n = 81)**	**Poor neurological outcome (n = 118)**	**Univariate analysis**		**Multivariate analysis**	
			**Odds ratio (95% CI)**	***P*** **-value**	**Odds ratio (95% CI)**	***P*** **-value**
Acute kidney injury, n (%)	29 (36)	56 (47)	0.664 (0.368, 1.197]	0.17		
Age, years	58 (49 to 71)	66 (53 to 77)	0.981 (0.963, 0.999)	0.04	0.963 (0.937, 0.990)	0.007
Weight, kgs	77 (70 to 90)	75 (65 to 85)	1.012 (0.993, 1.031)	0.24		
Male, n (%)	63 (78)	71 (60)	2.622 (1.341, 5.126)	0.005		
Witnessed CA	70 (87)	78 (66)	2.695 (1.283, 5.664)	0.009		
Bystander CPR, n (%)	60 (74)	52 (44)	2.823 (1.526, 5.224)	0.001	3.682 (1.522, 8.908)	0.004
Time to ROSC, min	13 (7 to 21)	18 (10 to 27)	0.980 (0.958, 1.002)	0.07	1.085 (1.009, 1.167)	0.03
Epinephrine, mg	3 (1 to 5)	5 (3 to 7)	0.846 (0.766, 0.935)	0.001	0.594 (0.439, 0.804)	0.001
Out- of-hospital CA	45 (56)	76 (64)	0.836 (0.465, 1.503)	0.55		
Non-cardiac origin CA	18 (22)	51 (43)	0.297 (0.150, 0.586)	<0.001		
VF/VT, n (%)	52 (64)	29 (25)	3.858 (2.925, 4.710)	<0.001	3.797 (2.922, 4.539)	<0.001
Infection during ICU stay, n (%)	49 (60)	74 (63)	1.165 (0.640, 2.121)	0.63		
IABP during ICU stay, n (%)	6 (7)	9 (8)	1.137 (0.388, 3.334)	0.82		
ECMO during ICU stay, n (%)	10 (12)	12 (10)	1.193 (0.483, 2.943)	0.70		
Shock during ICU stay, n (%)	36 (44)	67 (57)	0.531 (0.296, 0.950)	0.03	0.433 (0.187, 0.998)	0.049
Corticosteroid therapy, n (%)	10 (12)	19 (16)	0.720 (0.309, 1.677)	0.45		
Chronic heart failure, n (%)	20 (24)	37 (31)	0.502 (0.255, 0.989)	0.05		
Hypertension, n (%)	28 (35)	49 (42)	0.786 (0.433, 1.426)	0.43		
Coronary artery disease, n (%)	37 (46)	49 (42)	1.093 (0.612, 1.952)	0.76		
Diabetes, n (%)	16 (20)	26 (22)	0.705 (0.340, 1.462)	0.35		
COPD/asthma, n (%)	17 (21)	20 (17)	1.190 (0.574, 2.470)	0.64		
Neurological disease, n (%)	9 (11)	21 (18)	0.686 (0.296, 1.589)	0.38		
Chronic renal disease, n (%)	11 (14)	19 (16)	0.813 (0.358, 1.846)	0.62		
Liver cirrhosis, n (%)	2 (2)	9 (8)	0.358 (0.075, 1.704)	0.19		
Vasopressor therapy, n (%)	50 (62)	84 (71)	0.628 (0.342, 1.151)	0.13		
Duration of vasopressor therapy, days	4 (2 to 5)	3 (2 to 5)	1.032 (0.918, 1.159)	.060		
Cumulative vasopressor dose, mcg	10.5 (3.8, 28.8)	31.0 (11.1, 101.8)	0.992 (0.986, 0.999)	0.02		
Dobutamine therapy, n (%)	39 (48)	55 (47)	0.900 (0.650, 1.246)	0.53		
Cumulative dobutamine dose, mcg	964 (274 to 1978)	1746 (722 to 3378)	1.002 (1.001, 1.009)	0.04		
Duration of MV, days	3 (2 to 5)	3 (2 to 5)	1.052 (0.975, 1.136)	0.19		
CRRT, n (%)	11 (14)	13 (58)	1.491 (0.631, 3.525)	0.36		
Lactate on admission, mEq/L	2.4 (1.6, 3.8)	3.8 (2.1, 6.8)	0.844 (0.758, 0.940)	0.002	0.864 (0.746, 0.999)	0.049
Fluid balance at 48 h, mL	3714 (1867 to 5970)	4197 (2575 to 6729)	1.199 (0.875, 1.342)*	0.53		
CrCl on day 1, mL/min	54 (23 to 92)	33 (11 to 78)	1.002 (0.998, 1.007)	0.32		

Results are expressed as median (IQR) or number (%). *Per liter of fluid balance. CA, cardiac arrest; CPR, cardiopulmonary resuscitation; ROSC, return of spontaneous circulation; VF/VT, ventricular fibrillation/ventricular tachycardia; COPD, chronic obstructive pulmonary disease; IABP, intra-aortic balloon pump counterpulsation; ECMO, extracorporeal membrane oxygenation; MV, mechanical ventilation; CVVH, continuous veno-venous hemofiltration; CrCl, creatinine clearance.

## Discussion

AKI developed in 43% of patients resuscitated after CA and more than 75% of these episodes occurred within 3 days after CA. Almost one third of these patients with AKI eventually needed CRRT. Patients who developed AKI not only took longer for ROSC, but also more frequently had pre-existing cardiac and renal disease, had more severe hemodynamic alterations, a more positive fluid balance and received more nephrotoxic agents. However, although patients with AKI had increased hospital mortality, the development of AKI was not associated with significantly different rates of survival with a favourable neurological outcome.

Other studies have reported lower rates of AKI after CA, ranging from 12 to 43% [10,24,25]. These differences may be due to using different definitions of AKI, which in some studies included a fixed cutoff for sCr (>1.4 mg/dL) [24,25] rather than relative changes in creatinine from baseline values and UO. Moreover, some of the previous studies included only OHCA patients, whereas IHCA may be associated with different risk factors for AKI and for poor outcome [6,11,12,24]. Also, TH was used inconsistently in the other studies [9,11,12] and the time course of AKI was not reported.

As expected, AKI was more common in the presence of pre-existing renal dysfunction. This has been reported in other studies, in which conditions such as congestive heart failure or diabetes, which are associated with reduced renal function, were independent predictors of AKI after CA [6,24]. The association between IHCA and the occurrence of AKI may also reflect pre-existing disease leading to hospital admission and that could have predisposed the kidney to further injury after CA [26]. Older age was associated with an increased risk of AKI, as also suggested in a study by Yanta *et al*. [12], likely because of the physiological decrease in the renal reserve. The presence of shock was an independent predictor of AKI, as also reported in another study, which showed that AKI occurred in under 10% of hemodynamically stable CA patients [10]. Acute tubular necrosis is uncommon in postmortem studies of patients dying of shock [27]. A study by Chang *et al*. suggests that glomerular barrier dysfunction and vascular hyperpermeability may also play an important role in the pathogenesis of post-CA renal dysfunction [28]. Patients with AKI also received greater cumulative epinephrine doses than others; vasoconstrictors can induce alterations in intra-renal hemodynamics [29] and epinephrine may worsen the function of all organs, as suggested by alterations in cerebral microcirculation and lung exchange or increased post-resuscitation myocardial dysfunction observed in porcine models of CA [30]. Although the pathogenesis of post-resuscitation shock is multifactorial and encompasses systemic inflammatory response, myocardial stunning or adrenal insufficiency, our findings highlight that post-resuscitation disease plays a crucial role in the development of AKI and that identification of patients at risk could promote the development of nephro-protective strategies (for example, antioxidants, ARBs, phosphodiesterase inhibitors) in order to reduce AKI occurrence in such patients.

The association between high fluid balance at 48 h after arrest and AKI is also an important finding. Indeed, on one hand the maintenance of adequate circulating blood volume is essential to preserve renal perfusion: Adler *et al*. showed that fluid therapy guided by volumetric and arterial waveform-derived variables could reduce the incidence of AKI in patients with cardiogenic shock after CA [11]. By contrast, there is growing evidence that liberal fluid therapy might have detrimental effects in critically ill patients and that the type of fluid could have significant effects on renal injury [31]. In our study, the proportion of patients receiving HES was similar in patients who developed AKI and those who did not, but even chloride-rich crystalloid solutions may increase the risk of AKI [32]. Importantly, fluid resuscitation may also potentially dilute sCr and delay the diagnosis of AKI. This was elegantly shown by Pickering *et al*. [33] in a study evaluating 49 CA patients; AKI was observed in 6 patients demonstrating only minor changes in sCr but significantly increased biomarkers of renal injury. The changes in sCr among CA survivors may also be related to a temporary reduction of creatinine production (that is, due to the decreased non-enzymatic conversion of creatine to creatinine induced by the use of hypothermia or due to immobilization) [34,35]. The clinical consequence is that sCr alone may underestimate the occurrence and severity of AKI; thus, the UO evaluation is of great importance in this setting as it was shown to adequately identify AKI in most of these patients without sCr changes after CA.

As all patients were treated with TH after CA, we could not evaluate any protective role of cooling on renal function. Some studies showed that TH had beneficial effects on renal function after experimental CA or renal transplantation by reducing the extent of ischemia-reperfusion renal injury [36,37]. In a meta-analysis that also included post-cardiac surgery patients, Susantitaphong *et al*. suggested that TH did not protect renal function, although lower targets of cooling temperature were associated with lower risk of AKI [38]. In CA patients, two studies found that hypothermia was associated with an increased risk of AKI and a longer period to recover renal function [12,39]. Cooling may not per se be deleterious to renal function but may reflect more severe and longer duration of ischemic injury. Further studies are needed to better define the role of TH on renal protection after CA.

For the first time, we evaluated the prevalence and the impact of ARC in patients surviving CA; 19% of these patients had ARC on ICU admission, with more than 40% surviving with good neurological recovery at 3 months after arrest. ARC is present in 50 to 65% of the general ICU population on admission, and usually persists over subsequent days [13,40]. In our study, although ARC on admission was independently associated with a lower risk of developing AKI, it had no impact on neurological outcome. However, ARC may have other unwanted effects, such as increased elimination of antibiotics, which may expose patients to insufficient drug concentrations in the presence of infection, increased elimination of sedative agents that may complicate temperature control in this setting, and other difficulties in appropriate drug dosing, for example, in the case of seizures [13,41].

Our data provide no evidence that development of AKI contributes to poor neurological outcome in the setting of CA. By contrast, Hasper *et al*. report that the development of AKI is associated with a lower probability of favorable neurological outcome (72.5% versus 47.1%, *P* = 0.013) [9]. All other studies on the impact of AKI in CA patients evaluated only mortality. As such, Yanta *et al*., in a large cohort of 311 patients with OHCA, found that AKI had no impact on hospital survival [12]. Roberts *et al*. reported that extra-cerebral organ dysfunction was common after CA but only cardiovascular dysfunction and altered gas exchange were associated with outcome [4]. It is very difficult to compare these studies, because the measured outcomes were diverse (that is, hospital survival, favorable neurological outcome at hospital discharge and favorable neurological outcome at 3 months), the number of patients treated with TH was different, and so was the proportion of patients with OHCA. Importantly, as a decision to withdraw therapy is made in a number of patients with extended post-CA brain injury, regardless of the development of AKI, it is not possible to assess the impact of AKI on such decisions. Other factors, such as time to ROSC, total amount of epinephrine, presentation rhythm, bystander CPR and age, had a greater impact on neurological outcome than the development of AKI.

The study has some limitations. First, as a retrospective study, we may have missed some confounders that could influence the development of AKI. However, we collected a large amount of clinical data from the medical files, as well as data related to CA characteristics and the administration of potential nephrotoxic agents to minimize this bias. Of course, the generalizability of these findings may be limited because of different therapeutic strategies in the management of CA patients within other centers, which may influence both AKI occurrence and overall outcome. Second, during the four years of study enrolment, management of CA treatment may have changed somewhat [42], although this is unlikely to have influenced the occurrence of AKI over this relatively short period. Third, we did not assess the occurrence of rhabdomyolysis; although its incidence after CA remains largely unknown, elevation in creatine phosphokinase levels may contribute to severe AKI in this setting [43]. Moreover, rhabdomyolysis could be associated with marked creatinine release from skeletal muscle, which may theoretically influence the course of sCr. Fourth, we did not record other findings from urine analysis, such as proteinuria or specific gravity, although these have been associated with patient outcome after CA [28]. Also, we did not use the standard UO criteria for AKI stage 1 (that is <0.5 mL/kg/h for 6 h) and this may have underestimated the occurrence of AKI stage 2 and/or stage 3 in our population. Fifth, the number of patients with poor neurological outcome surviving at hospital discharge was quite limited and was significantly influenced by the short-term survival. Thus, it is difficult to make a conclusion on the influence of AKI on neurological recovery, given the analysis is dominated by death. However, no more than 10% of CA patients with post-anoxic severe disability or vegetative status are discharged alive from the hospital and only a few of them have improvement of their long-term neurological status [44]. Sixth, for in-hospital CA, we used sCr at hospital admission as the baseline value and this may have been influenced by the underlying reason for admission. Also, we did not adjust our results using different scores for comorbidity, such as the Charlson, Davies or Khan indices, which have been shown to predict outcome in patients with end-stage renal disease [45]. Nevertheless, the predictive value of such scores in patients with CA remains controversial [46]. Finally, we did not assess long-term renal function in CA survivors. However, Chua *et al*. showed that creatinine levels on discharge were similar to baseline values in such patients, and that none of those being treated with CRRT during their ICU stay needed long-term hemodialysis thereafter [10].

## Conclusions

In this cohort, AKI occurred in almost half the patients who survived CA; however, it was not an independent prognostic factor for poor outcome. Age, epinephrine dose, cumulative fluid balance and presence of shock were independent predictors of the development of AKI in this population. ARC occurred in almost 20% of patients and was associated with reduced incidence of AKI. Renal failure had no clear correlation with poor neurological outcome.

## Key messages

- AKI is common in patients admitted after CA.
- Major risk-factors for AKI are older age, chronic renal disease, higher dose of epinephrine, in-hospital CA, presence of shock during the ICU stay, low creatinine clearance (CrCl) on admission and a high cumulative fluid balance at 48 h.
- AKI was not an independent predictor of poor 3-month neurological outcome.

## Abbreviations

ACEI: angiotensin-converting enzyme inhibitor
AKI: acute kidney injury
ARB: angiotensin II receptor blocker
ARC: augmented renal clearance
BAL: bronchoalveolar lavage
CA: cardiac arrest
CDC: Centers for Disease Control and Prevention
CKD: chronic kidney disease
CPC: cerebral performance category
CPR: cardiopulmonary resuscitation
CrCl: creatinine clearance
CRRT: continuous renal replacement therapy
ECMO: extracorporeal membrane oxygenation
EEG: electroencephalogram
GCS: Glasgow coma scale
HES: hydroxyethyl starch
IABP: intra-aortic balloon counterpulsation
IHCA: in-hospital cardiac arrest
MDRD: Modification of diet in renal disease
NSAID: non-steroidal anti-inflammatory drug
OHCA: out-of-hospital cardiac arrest
ROSC: return of spontaneous circulation
sCr: serum creatinine
TH: therapeutic hypothermia
UO: urine output
VF/VT: ventricular fibrillation/tachycardia
